# Brain glucose metabolism in patients with traumatic brain injury undergoing rehabilitation: a longitudinal 18F-FDG PET study

**DOI:** 10.3389/fneur.2025.1556427

**Published:** 2025-03-06

**Authors:** Valeria Pingue, Irene Bossert, Daniela D’Ambrosio, Antonio Nardone, Giuseppe Trifirò, Nicola Canessa, Diego Franciotta

**Affiliations:** ^1^Department of Clinical-Surgical, Diagnostic and Pediatric Sciences, University of Pavia, Pavia, Italy; ^2^Neurorehabilitation and Spinal Units of Pavia Institute, Istituti Clinici Scientifici Maugeri IRCCS, Pavia, Italy; ^3^Nuclear Medicine Unit, Istituti Clinici Scientifici Maugeri IRCCS, Pavia, Italy; ^4^Medical Physics Unit, Istituti Clinici ScientificiMaugeri SpA SB IRCCS, Pavia, Italy; ^5^IUSS Cognitive Neuroscience (ICON) Center, Scuola Universitaria Superiore IUSS, Pavia, Italy; ^6^Istituti Clinici Scientifici Maugeri IRCCS, Cognitive Neuroscience Laboratory of Pavia Institute, Pavia, Italy; ^7^UOM, Laboratory of Clinical Pathology, Department of Laboratories, APSS Santa Chiara Hospital, Trento, Italy

**Keywords:** traumatic brain injury, rehabilitation, PET scanning, neuroplasticity, voxel-wise analyses

## Abstract

**Background:**

Measuring 18F-FDG PET-detected brain glucose uptake provides reliable information on metabolic tissue abnormalities, cells dysfunction, and neurovascular changes after traumatic brain injury (TBI).

**Objectives:**

We aimed to study the relationship between post-traumatic brain glucose metabolism and functional outcomes in the so far unexplored field of longitudinally 18F-FDG PET-monitored patients undergoing rehabilitation after moderate-to-severe TBI.

**Methods:**

Fourteen patients consecutively admitted to our unit in the post-acute phase after TBI underwent 18F-FDG-PET scans performed before and 6 months after inpatient rehabilitation program. The Glasgow Coma Scale (GCS) for neurological status, and the Functional Independence Measure (FIM) plus the Glasgow Outcome Scale-Extended (GOSE) scales for the rehabilitation outcome, were applied on admission and discharge. Voxel-wise analyses were performed, with the Statistical Parametric Mapping (SPM12) software, to investigate pre- vs. post-rehabilitation changes of brain metabolism, and their relationships with clinical indices.

**Results:**

In the whole sample, 18F-FDG uptake significantly increased in the following five regions that were hypometabolic before rehabilitation: inferior frontal gyrus bilaterally, alongside right precentral gyrus, inferior parietal lobule, and cerebellum. However, only for the right precentral gyrus the median voxel peak-value at baseline resulted a significant predictor of both cognitive (FIM cognitive subscale, *p* = 0.012), and functional (GOS-E, *p* = 0.02; post- vs. pre-treatment GOS-E difference, *p* = 0.009) improvements. ROC curve analysis showed that a peak voxel-value of 1.7998 was the optimal cut-off for favorable rehabilitation outcome. Unfavorable functional outcomes were predicted by increased 18F-FDG uptake in the inferior frontal gyrus (GOS-E, *p* = 0.032) and precentral gyrus (FIM cognitive subscale, *p* = 0.017; GOS-E, *p* = 0.015).

**Conclusion:**

This proof-of-principle study enlightens the metabolic changes occurring in moderate-to-severe TBI course. Notably, such changes preferentially involve definite frontal brain areas regardless of TBI localization and entity. These findings pave the way for further studies with translational purposes.

## Introduction

Traumatic brain injury (TBI) is defined as an alteration of the brain function leading to neurological, behavioral, and cognitive impairment as a result of biomechanical insults (1). With an estimated annual incidence rate between 134 and 618 per 100,000 persons (2), TBI is the most common cause of neurological disorders (3), and of death and disability in children and young adults worldwide (4, 5).

Brain damage after TBI can follow a combination of primary mechanical and delayed secondary injury (6). Concussive impacts immediately produce abnormal ionic fluxes that result in imbalanced ionic concentrations in the brain tissue, along with an indiscriminate release of excitatory neurotransmitters (7). These acute abnormalities are followed by hypometabolic states and abnormal neuronal functioning (due to axonal and mitochondrial damage), decreased cerebral blood flow (CBF) (7), and blood–brain barrier (BBB) dysfunction (8). CBF reduction leaves glycolysis as the main pathway to satisfy metabolic requirements during TBI acute and post-acute phases (7). Two-deoxy-2-(18F) fluoro-D-glucose (18F-FDG) is a radiotracer developed for the study of glucose metabolism and, specifically, the rate of cellular glycolysis (9). To date, widespread and consistent evidence has confirmed the reliability of 18F-FDG PET in assessing brain metabolic alterations due to both pathophysiological cerebral tissue and neurovascular changes after TBI (10–12).

Indeed, 18F-FDG PET studies on this topic have demonstrated that both the extent of metabolic decrease, and length of metabolic recovery of hypometabolism are related to TBI severity (10–14). In some cases, the hypometabolism in specific brain regions has been correlated with cognitive and behavioral dysfunction (10, 13, 15). A recent study on patients with cognitive impairment after TBI, reported decreased metabolic activity in the prefrontal cortex, and increased 18F-FDG uptake in the limbic system, which were associated with chronic symptoms of depression (15). However, due to heterogeneous clinical presentation of TBI, no well-defined metabolic patterns capable to predict TBI outcome have been identified so far (16). Moreover, in addition to characterizing hypometabolic patterns, there is the need to establish whether 18FDG PET can track changes in glucose metabolism indicative of adaptive, or maladaptive reshape of brain damage (17). This could help clinicians to predict the evolution of the disease and, in turn, middle-/long-term complications.

Based on the hypothesis that the topography and temporal evolution of metabolic changes of distinctive brain areas could help define prognosis and predict rehabilitation outcome in TBI, we performed a prospective, longitudinal study on TBI patients who underwent brain 18F-FDG PET scans and multiple functional scale evaluations before and after a standardized rehabilitation program.

## Materials and methods

### Study design and population

**Figure 1:**
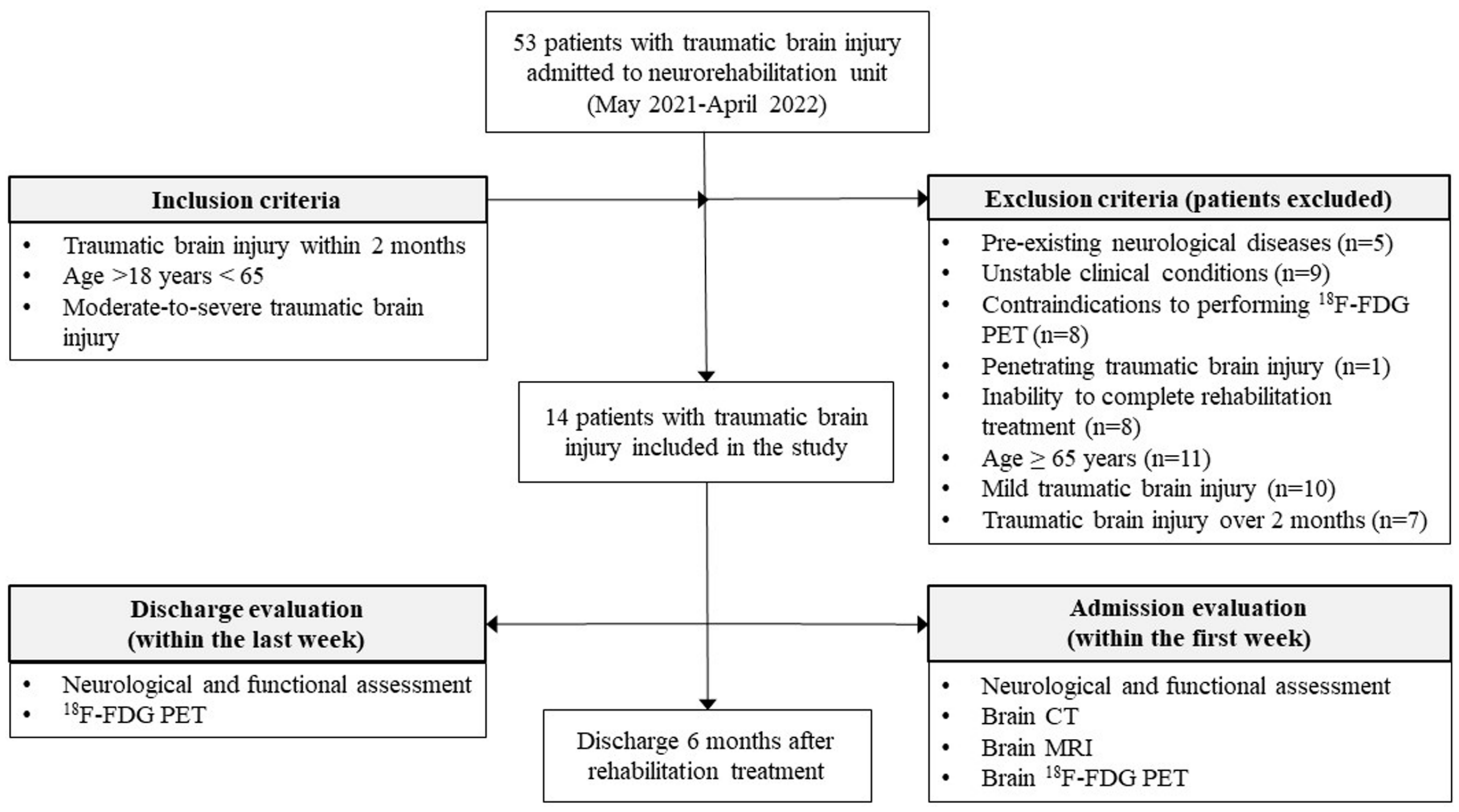
Algorithm of the study design.

In this single-center prospective study, we included 14 patients (mean age, 44.3 ± 14.8; male/female ratio, 2.5:1.0) with moderate-to-severe TBI, consecutively admitted to the Neurorehabilitation Unit of the ICS Maugeri Institute of Pavia (IRCCS) between May 21, 2021, and April 23, 2022. The inclusion criteria were: (1) age ≥ 18 years; (2) admission for a rehabilitation-based TBI treatment program within 2 months after TBI; (3) moderate [Glasgow Coma Scale (GCS) score, 9–12] to severe (GCS score, 3–8) TBI on admission. We excluded patients with pre-existing neurological diseases, age ≥ 65 years, unstable neurological, or non-neurological clinical conditions (e.g., behavioral disorders, uncontrolled status epilepticus, or sepsis), presence of penetrating TBI, or inability to complete rehabilitation treatment (e.g., death during hospitalization) (see Figure 1).

Following the acute event and post-acute hospitalization, all patients underwent a 6-month standard neurorehabilitation program, consisting of treatment cycles performed 6 days per week. These treatments included nursing assistance, physiotherapy, occupational therapy, speech therapy, cognitive training, nutrition assistance, as well as neuropsychological and social support.

The study design complied with the ethical guidelines of the Declaration of Helsinki, and was approved by the local Ethics Committee of ICS Maugeri (#2532 CE/2021). All participants, or their authorized representatives, signed a written informed consent.

### Variables and measurements

We collected the following variables: sex, age at occurrence of injury, neurological and functional evaluation, as well as injury characteristics on admission, and, particularly, the presence of focal damage (e.g., subdural hematoma, epidural hematoma, cerebral contusion) on brain CT, and/or of diffuse axonal injury on brain MRI, with the associated neurosurgical procedures (craniotomy or craniectomy). All patients underwent clinical and instrumental examinations within 1 week of admission to the Unit. 18F-FDG PET acquisitions were performed at baseline (1 week after admission), and within 1 week of discharge, respectively. GCS is a standardized system for evaluating the degree of neurological impairment and level of consciousness in all kinds of brain injury, which is classified as severe (3-to-8 scores), moderate (9-to-12), or mild (13-to-15) (18). Functional and global outcomes were evaluated, across baseline and discharge time points, by comparing pre- and post-scores of two scales, namely the Functional Independence Measure (FIM), which measures disability by tracking progress in functional status (19), and the Glasgow Outcome Scale-Extended (GOS-E), which evaluates the global outcome in TBI patients (20). FIM scores are calculated through 13 motor (FIM-m) and 5 cognitive (FIM-c) items in terms of burden of care. Each item is graded on a 1-to-7 scale, based on the level of independence (1 = total assistance required, 7 = complete independence). GOS-E scores can be dichotomized as favorable (scores, 4–8), or unfavorable (scores, 1–3) outcome in relation with patients’ dependence on others and on community reintegration (21). These scales are routinely used in clinical practice to evaluate rehabilitation outcomes.

### 18F-FDG PET image acquisition and reconstruction

All patients underwent pre- and post-rehabilitation 18F-FDG PET scan acquisitions (GE Discovery 690 PET), in accordance with the guidelines of the European Association of Nuclear Medicine (22). We collected static emission images 45-min after injecting 2, 5 MBq/Kg of 18F-FDG in fasting patients. This post-injection time interval enables an equal distribution of the tracer across the entire brain, with negligible blood flow-dependent differences, and an optimal signal-to-noise ratio (23). Scan acquisition lasted 15 min. Uniform reconstruction protocols were applied. In particular, we used both ordered subset-expectation maximization algorithms and CT attenuation-based correction procedures. A quality control process was performed to check for major artefacts in raw images, including defective image uniformity and orientation, or attenuation correction due to mismatches between CT and PET images.

### 18F-FDG PET image pre-processing and correlated statistical analysis

We used the Statistical Parametrical Mapping (SPM12) software,^1^ as implemented in MATLAB (Mathworks Inc., Sherborn, MA, United States), to perform a standard pre-processing of PET scans. Each image was first normalized to a standardized 18F-FDG PET template registered to the Montreal Neurological Institute standard space (24), using the default SPM12 bounding-box, resampled at an isotropic voxel size of 2 mm, and spatially smoothed with an 8 mm isotropic 3D Gaussian FWHM kernel. The 18F-FDG PET template has been reported to ensure high normalization accuracy, and to reduce noise-related random effects (24). To account for the between-subject 18F-FDG uptake variability (25), each image was proportionally scaled to its global mean (26). This approach enables higher signal-to-noise ratio, in comparison with the other available scaling methods (e.g., cerebellar reference area) (27).

We used paired-sample t-testto investigate longitudinal changes by comparing brain metabolism across admission and discharge time points in the whole sample. We reported as statistically significant only the voxels surviving a statistical threshold of *p* < 0.05, which was corrected for multiple comparisons based on cluster extent using topological false discovery rate, with *p* < 0.005 at the voxel level. Toolbox rex^2^ was used to extract, from the resulting clusters, the mean 18F-FDG metabolism levels of each patient’s pre- and post-treatment statistical maps, for subsequent correlation analysis matched with clinical measures.

### Statistical analysis

The reported values were expressed as median and interquartile range (IQR), or absolute frequency and percentage, measured before (T0) and after (T1) the rehabilitation program, and calculated as absolute variations over baseline values (Δ = T1−T0). Data were tested for normality of distribution via Shapiro–Wilk test, and log-transformed when needed to correct for skewness. Comparisons of neurological and functional measures across admission and discharge were performed through Wilcoxon signed-rank test. We used Spearman’s correlation index to identify relationships between strength of metabolism in the brain regions showing significant pre- vs. post-changes, and rehabilitation outcomes.

Analysis through receiver operating characteristic (ROC) curves, and area under the curve (AUC), was performed to assess the accuracy of 18F-FDG PET uptake values in the right precentral gyrus for distinguishing between patients with favorable (GOS-E scores 4–8) vs. unfavorable (GOSE scores 1–3) functional outcome (21).

Finally, a multiple linear regression analysis was carried out to test the potential predictive role of 18F-FDG uptake values, as measured in the brain regions showing significant pre- vs. post-changes possibly associated with functional outcome. The multiple model included a combination of independent variables, including age, sex, severity of TBI, laterality of contusion. Linearity and univariate analyses between dependent and independent variables were checked. Statistical analyses were performed using IBM SPSS Statistics version 21 (Somers, NY, United States). Threshold for statistical significance was set at *p* < 0.05.

## Results

Table 1 summarizes the demographic and clinical features of the 14 enrolled patients.

**Table 1:** Clinical-demographic features of the 14 patients with traumatic brain injury.

Age, years	44.3 (±14.8)
Gender, male/female	10/4
Interval between injury and inpatient rehabilitation (days)	34.3 (±10.0)
Focal brain damage	14 (100.0)
Diffuse axonal injury	12 (85.7)
Decompressive procedures	4 (28.6)

Values are expressed as mean and standard deviation (SD), or absolute number and percentage.

Structural focal damage, detected in all patients, mainly involved frontal (14 patients), and temporal lobes (10 patients), without a significant hemispheric prevalence (*p* = 0.788). Twelve patients displayed both focal and diffuse lesions on brain CT and MRI.

All the enrolled patients completed the intensive rehabilitation program, and achieved significant neurological and functional improvement (Table 2).

**Table 2:** Neurological and functional assessment of patients with traumatic brain injury on admission and on discharge from rehabilitation unit.

	Admission (T0)	Discharge (T1)	*P*
Glasgow Coma Scale score	10 (8.5–12)	15 (12.5–15)	0.002
Functional Independence Measure total score	18.5 (18–25)	75 (24–101)	0.001
Functional Independence Measure motor subscale	13.5 (13–14)	48 (15–85)	0.003
Functional Independence Measure cognitive subscale	5 (5–11)	24 (10.5–28)	0.001
Glasgow Outcome Scale-Extended	2.5 (2–3)	4 (3–4)	0.008

Values are expressed as median and interquartile range (IQR). Comparison between T0 and T1 was performed by Wilcoxon paired-sample test.

**Figure 2:**
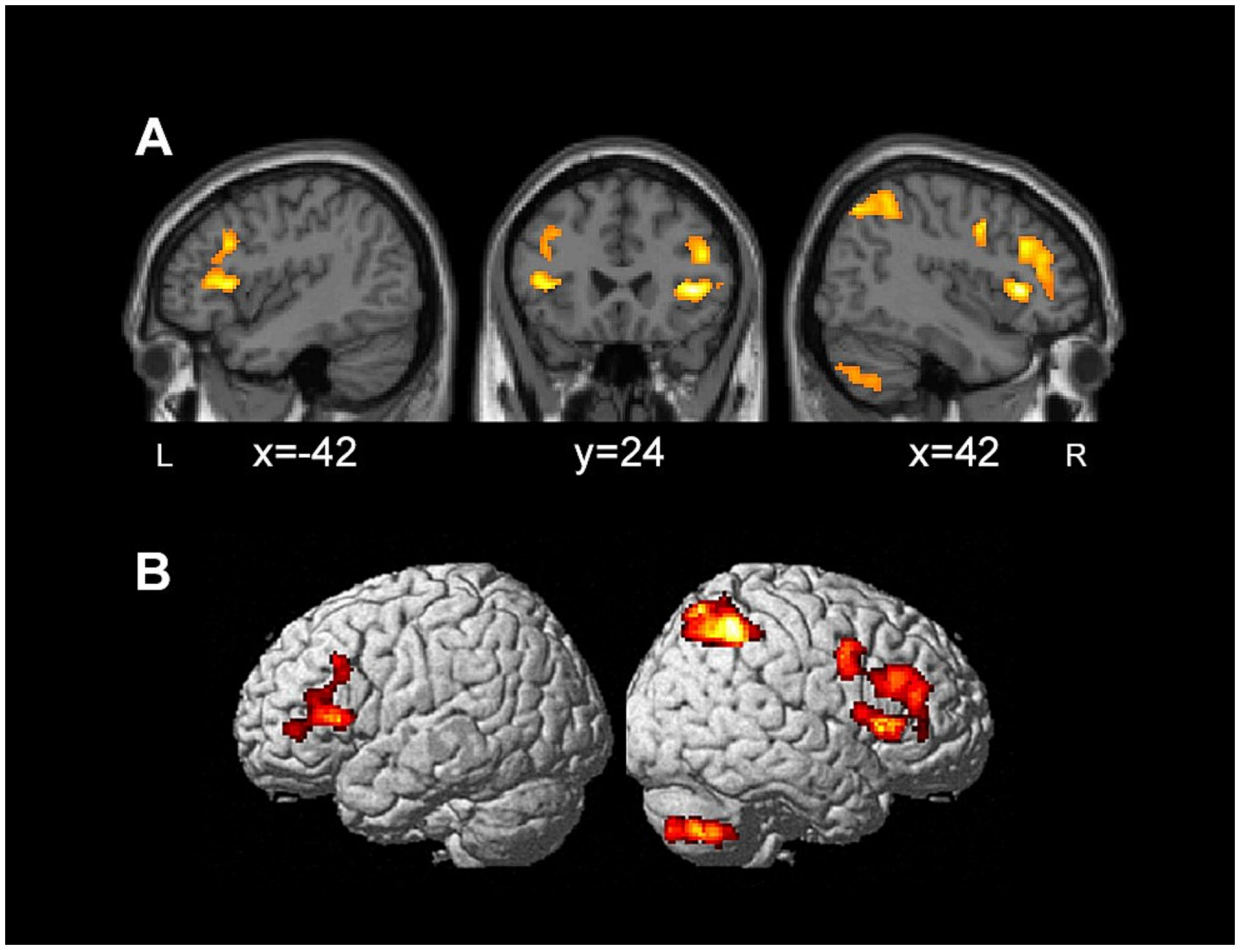
Brain regions with increased 18F-FDG PET glucose metabolism in patients with traumatic brain injury after rehabilitation, displayed on sagittal and coronal sections **(A)**, as well as 3D renders **(B)**.

Whole-brain comparisons of pre- vs. post-rehabilitation time points highlighted five hypometabolic clusters in which 18F-FDG uptake significantly increased on discharge, namely, the inferior frontal gyrus in both the hemispheres, alongside the precentral gyrus, inferior parietal lobule and cerebellum in the right hemisphere (Table 3; Figure 2). We considered these clusters as “Regions-of-Interest” (ROI) for subsequent correlation analysis between pre- vs. post-treatment metabolic and behavioral changes.

**Table 3:** Brain regions with 18F-FDG PET-assessed increase of glucose metabolism in patients with traumatic brain injury.

Brain regions	Peak^*^ coordinates (*x*, *y*, *z*)	Extent of peak voxel (mm^3^)	Z max	Δ Met.	Cluster-corrected *p*-value
Right inferior frontal gyrus	42, 24, 4	515	7.33	0.0459 (0.0114–0.1266)	0.037
Right precentral gyrus	48, 6, 44	786	6.75	0.1198 (0.0368–0.2348)	0.010
Right inferior parietal lobule	50, −48, 50	1,072	6.54	0.1470 (0.0589–0.2655)	0.003
Left inferior frontal gyrus	−42, 22, 8	573	5.86	0.1031 (0.0354–0.1382)	0.032
Right cerebellum	52, −60, −44	486	4.86	0.2608 (0.1272–0.3548)	0.038

^*^Coordinates at the peak voxel in each cluster based on the Montreal Neurological Institute atlas.

Δ Met, variation of metabolic pattern measured in peak voxel pre- vs post-rehabilitation program (median values and IQR).

**Figure 3:**
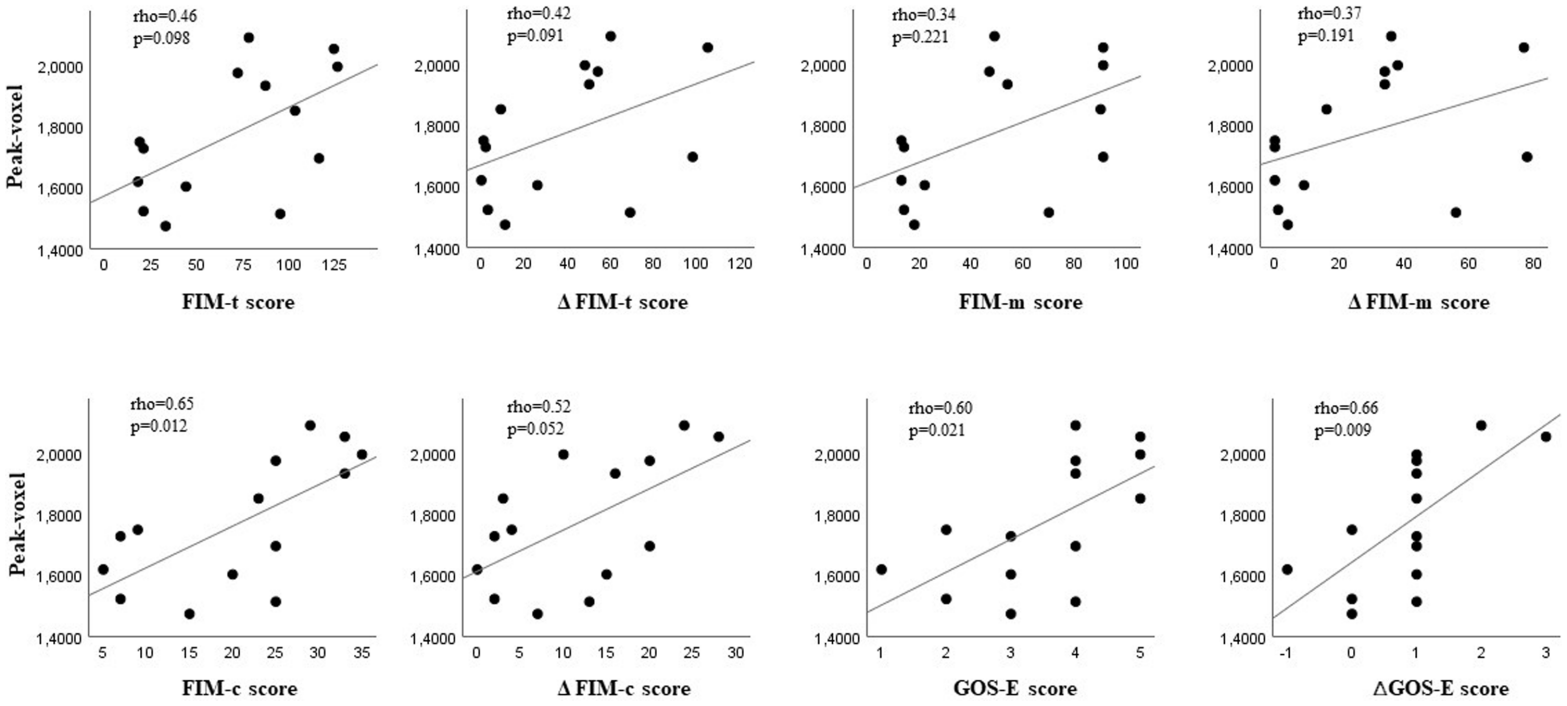
Basal glucose metabolism in the right precentral gyrus and functional outcome in patients with traumatic brain injury undergoing rehabilitation. Correlations between basal brain 18F-FDG PET-detected glucose uptake values in the right precentral gyrus and functional outcome on discharge, calculated as total scores and as post- vs. pre-treatment score differences on the following scales: Functional Independence Measure (FIM), FIM motor subscale (FIM-m), FIM cognitive subscale (FIM-c), and Glasgow Outcome Scale-Extended (GOS-E).

We therefore hypothesized a possible relationship between the increase of glucose consumption in these areas, and the improvement in rehabilitation outcome, as measured with the functional scales. Through a cross-sectional analysis, we first correlated the pre-rehabilitation 18F-FDG uptake values of the five ROI with the functional outcome of TBI patients on discharge. Only the change in glucose uptake within the right precentral gyrus showed a significant positive correlation with better functional recovery on discharge, as measured with the FIM cognitive subscale (rho = 0.65, *p* = 0.012), and the GOS-E scores (rho = 0.60, *p* = 0.021) measured at baseline. Post- vs. pre-treatment difference was also significant for the GOS-E (rho = 0.66, *p* = 0.009) (Figure 3).

We further checked the cross-sectional analysis for potential confounding variables. The linear regression model quantified whether, and to what extent, baseline 18F-FDG uptake in the right precentral gyrus predicted functional outcome (Table 4).

**Table 4:** Multiple linear regression analysis of clinical-demographic and 18F-FDG PET scan variables on admission as predictors of rehabilitation outcome.

Independent variables	FIM tot (R^2^ = 0.812)		FIM-m (R^2^ = 0.767)		FIM-c (R^2^ = 0.892)		GOS-E (R^2^ = 0.807)	
	β	*p*	β	*p*	β	*p*	β	*p*
Sex (M = 0, *F* = 1)	−0.32	0.242	−0.435	0.169	−0.06	0.73	−0.40	0.16
Age (years)	−0.14	0.516	−0.163	0.500	−0.02	0.89	−0.05	0.79
GCS score	0.58	**0.030**	0.618	**0.038**	0.45	**0.03**	0.50	0.06
Right hemisphere contusion	0.20	0.444	0.120	0.687	0.35	0.11	0.23	0.40
Left hemisphere contusion	−0.10	0.717	−0.222	0.483	0.08	0.70	−0.28	0.33
Glucose metabolism of right precentral gyrus	0.47	**0.033**	0.412	0.079	0.60	**0.003**	0.55	**0.01**

FIM-tot, Functional Independence Measure total scale; FIM-m, Functional Independence Measure motor subscale; FIM-c, Functional Independence Measure cognitive subscale; GCS, Glasgow Coma Scale; GOS-E, Glasgow Outcome Scale-Extended. Significant *p* values in bold.

18F-FDG uptake in the right precentral gyrus before the rehabilitation program emerged as the strongest independent predictor of the distinct facets of recovery on discharge, measured with FIM total scale (*β* = 0.474, *p* = 0.033), FIM cognitive subscale (*β* = 0.606, *p* = 0.003), and GOS-E (*β* = 0.556, *p* = 0.018). As expected, GCS measured brain damage severity at T0 emerged as the main predictor of functional outcome, independently from the other variables included in the regression model. Univariate analyses confirmed that the variables reported in Table 4 were independently related to outcome.

ROC curve analysis showed that a peak voxel value of 1.799 in the right precentral gyrus identified patients with favorable outcome assessed with GOS-E on discharge, with a sensitivity of 75%, and a specificity of 100% (AUC = 0.854, CI 95% 0.640–0.1000, *p* = 0.028) (Supplementary Figure S1).

**Figure 4:**
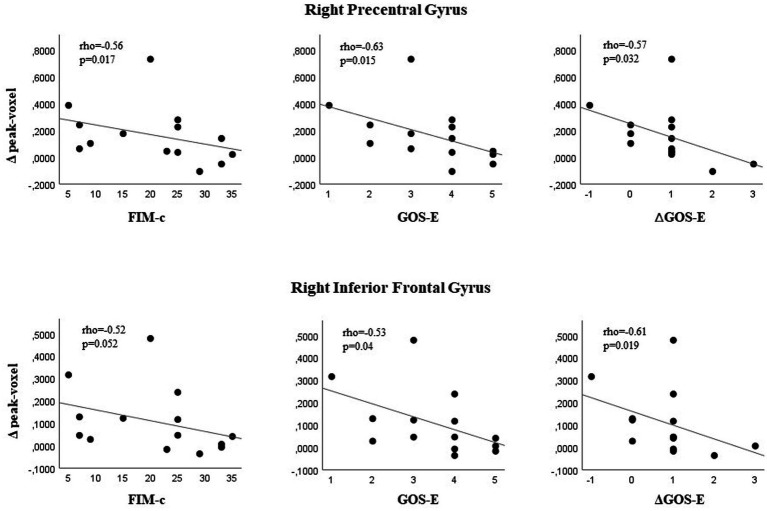
Glucose metabolic changes in the right precentral/inferior frontal gyrus and functional outcome in patients with traumatic brain injury undergoing rehabilitation. Correlations between pre- vs. post-treatment brain 18F-FDG PET-detected glucose uptake values in the right precentral/inferior frontal gyrus and functional outcome on discharge, calculated as scores, total scores and as post- vs. pre-treatment score differences on Functional Independence Measure cognitive subscale (FIM-c), and Glasgow Outcome Scale-Extended (GOS-E).

Finally, we searched for a relationship between metabolic changes (post- vs. pre-treatment increase of glucose metabolism) in the five ROI, and functional outcome at discharge. The increase of 18F-FDG uptake in the right inferior frontal gyrus and right precentral gyrus was significantly associated with worse functional outcome scores on discharge (Supplementary Table S1). Namely, the post- vs. pre-treatment variations of glucose metabolism in the right precentral gyrus were significantly correlated with poor outcome, as measured with the FIM cognitive subscale (rho = −0.56, *p* = 0.017), and GOS-E score (rho = −0.63, *p* = 0.015). Post- vs. pre-treatment difference was also significant on the GOS-E (rho = −0.61, *p* = 0.019). Increased 18F-FDG uptake in the right inferior frontal gyrus was associated with worse scores on the GOS-E (rho = −0.57, *p* = 0.032), as well as its post- vs. pre-treatment difference (rho = −0.53, *p* = 0.04) (Figure 4).

## Discussion

In this longitudinal study we used 18F-FDG PET, before and after intensive inpatient rehabilitation in the post-acute phase of moderate-to-severe TBI, to examine the relationship between variations in brain glucose metabolism and functional outcome. Three main findings of our study are notable. First, voxel-wise analysis highlighted a significant improvement of 18-FDG uptake after the rehabilitation program in only five brain regions that were hypometabolic at the baseline. Second, among these areas, only in the right precentral gyrus the higher baseline levels of 18F-FDG uptake were associated with functional recovery in terms of disability. Third, surprisingly, glucose metabolism on discharge increased in the right inferior frontal gyrus and in precentral gyrus in patients with worse outcome scores on the FIM cognitive subscale and the GOS-E scale, among all the included patients, who showed post-rehabilitation functional improvement.

This unexpected finding suggests that an increase of glucose consumption in specific brain areas might entail different prognostic evaluations, namely, prediction of favorable outcome when detected before rehabilitation, or a less degree of functional improvement when detected 6 months later. This could be possibly explained by distinct processes equally sustained by increase in glucose consumption. Indeed, it is likely that, following a traumatic injury, the brain enters a sub-acute phase of enhanced plasticity, requiring energy supply that might ultimately foster recovery (28, 29). However, the brain circuitry can be reshaped in either adaptive, or maladaptive ways (28). In particular, persistent and low-grade neuroinflammation with relatively high tissue metabolic demands can interfere with the processes promoting recovery, thus facilitating neurodegeneration, and sustaining functional and cognitive deficits (30). Glucose hypometabolism, as measured with 18F-FDG PET, has been also correlated with chronic sequelae in TBI, typically involving cognitive and memory domains (8, 13, 15, 31). More recently, post-concussion chronic cognitive symptoms have been associated with decreased glucose consumption in the prefrontal cortex (15). The present results provide the first evidence that changes in glucose metabolism in frontal and pre-frontal gyrus could predict functional outcome in the post-acute phase of TBI.

In the attempt of quantifying the degree of hypometabolism for translational purposes, we showed that 18F-FDG PET voxel values above a given threshold (i.e., 1.799), detected on admission and before the rehabilitation program in the right precentral gyrus, might represent an early prognostic biomarker of good functional and rehabilitation outcome, independently from age, side of contusion, and severity of brain injury.

The prefrontal cortex plays a key role in processes like planning of voluntary movements, particularly in concert with selective structures of the cortical motor network, such as the rostral supplementary motor and lateral premotor areas (32, 33). Moreover, recent studies have highlighted its key role in the attention network, through the top-down cognitive control of sensory processing, and in goal-directed behavior (34). Therefore, the extent of structural and functional alterations in these prefrontal areas could possibly predict cognitive impairment after TBI (35, 36), but, in accordance with our data, may also have a role in functional recovery. This finding supports the hypothesis that the frontal lobes represent an ideal target of different types of rehabilitative strategies to maximise outcome (37). These might include, for instance, brain stimulation protocols that target the prefrontal cortex and especially the right precentral gyrus (38).

### Limitations

This study has limitations. In particular, the small sample size constraints both the generalizability of results and strength of the conclusions. Additionally, while assessing outcome within 6 months post-injury allows to capture the highest degree of recovery in patients with other acquired non-traumatic brain lesions (28), it may be poorly informative in those with TBI, who could continue to improve up to 1-year post-injury (39). Finally, the study protocol did not include in-depth cognitive evaluations through more appropriate neuropsychology battery tests, which could have enabled more accurate associations between glucose metabolism changes and neurocognitive improvements. The setting was also unfit for the impact evaluation of rehabilitation protocols on outcome.

## Conclusion

In conclusion, the present study provides preliminary data on the potential role of 18F-FDG PET as a useful prognostic tool for TBI patients undergoing rehabilitation courses. Voxel-wise analysis unveiled brain regions with high potential in terms of functional plasticity and neuronal network reorganization. In particular, these findings highlight a prefrontal 18F-FDG uptake, and its temporal evolution within the first months after injury, as a promising candidate of prognostic biomarker in TBI. Further studies with larger sample sizes and longer follow-up periods could help validate and expand upon these findings.

## Data Availability

The raw data supporting the conclusions of this article will be made available by the authors, without undue reservation.

## Footnotes

^1^ http://www.fil.ion.ucl.ac.uk/spm/software/SPM12/

^2^ https://www.nitrc.org/projects/rex/
