# Supplementary material for: Brain glucose metabolism in patients with traumatic brain injury undergoing rehabilitation: a longitudinal 18F-FDG PET study
Source: Front Neurol. 2025 Mar 6;16:1556427. doi: 10.3389/fneur.2025.1556427 (PMC11922693; doi:10.3389/fneur.2025.1556427)

**Supplementary table 1.** **Functional assessment of patients with traumatic brain injury on admission and discharge from rehabilitation unit**

| **Functional scales** | **Score on admission** | **Score on discharge** | ***P value*** |
| --- | --- | --- | --- |
| Functional Independence Measure total | 18.5 (18-25) | 75 (24-101) | 0.001 |
| Functional Independence Measure motor subscale | 13.5 (13-14) | 48 (15-85) | 0.003 |
| Functional Independence Measure cognitive subscale | 5 (5-11) | 24 (10.5-28) | 0.001 |
| Glasgow Outcome Scale-Extended | 2.5 (2-3) | 4 (3-4) | 0.008 |

*Values are expressed as median and interquartile range. Comparison between scores at T0 and T1 was performed by Wilcoxon paired-sample test.*

**Supplementary figure 1.** **ROC curve analysis**

Receiver operating characteristic (ROC) curve of 18F-FDG-PET voxel values detected in the right precentral gyrus on admission for rehabilitation treatment for differentiating patients with favourable outcome, as measured with Glasgow Outcome Scale-Extended (GOSE) scale at discharge
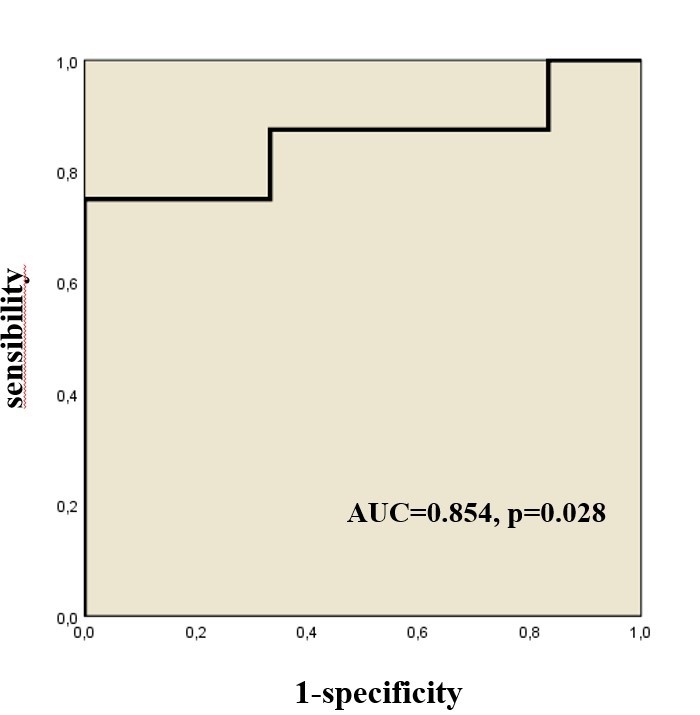

Supplement: Supplementary file 1 [file Table_1.docx]
